# Draft Genome Sequence of Two Salmonella enterica Subspecies *enterica* Serovar Minnesota Strains Harboring *Mcr-*1.1 Gene Isolated from Chicken Meat in Saudi Arabia

**DOI:** 10.1128/mra.00787-22

**Published:** 2022-10-04

**Authors:** Lenah E. Mukhtar, Ashwaq S. Alhamed, Mohammed I. Mujallad, Elaf A. Alshdokhi, Fahad M. Alreshoodi, Malfi S. Al Rashidy, Saleh I. AlAkeel, Sulaiman M. Alajel

**Affiliations:** a Antimicrobial Resistance Division, Reference Laboratory for Microbiology, Executive Department of Reference Laboratories, Research and Laboratories Sector, Saudi Food and Drug Authority (SFDA), Riyadh, Saudi Arabia; b Molecular Biology Division, Reference Laboratory for Microbiology, Executive Department of Reference Laboratories, Research and Laboratories Sector, Saudi Food and Drug Authority (SFDA), Riyadh, Saudi Arabia; c Microbial Identification Division, Reference Laboratory for Microbiology, Executive Department of Reference Laboratories, Research and Laboratories Sector, Saudi Food and Drug Authority (SFDA), Riyadh, Saudi Arabia; d Reference Laboratory for Microbiology, Executive Department of Reference Laboratories, Research and Laboratories Sector, Saudi Food and Drug Authority (SFDA), Riyadh, Saudi Arabia; University of Maryland School of Medicine

## Abstract

Salmonella has been reported as a high-priority pathogen by the World Health Organization. Here, we report the draft genome sequence of 2 Salmonella enterica serovar Minnesota strains isolated from chicken meat in Saudi Arabia (named SA49317 and SA49319) belonging to sequence type (ST) 548, revealing mobile colistin resistance (*mcr*)*-*1.1 gene for colistin resistance.

## ANNOUNCEMENT

Salmonella enterica serovar Minnesota is commonly associated with the supply chain of poultry meats (1). Colistin is considered as the last option for multidrug resistant Salmonella (2, 3). However, the heavy use of colistin in livestock led to the emergence of *mcr* genes (2, 3). In this report, we present the draft genome sequence of 2 colistin-resistant (carrying *mcr-*1.1) Salmonella enterica serovar Minnesota strains isolated, from chicken meat, collected from a local market in Riyadh, Saudi Arabia as a part of surveillance plan in 2020.

SA49317 and SA49319 strains were identified according to ISO 6579-1:2017 and ISO 6579-3:2014 methodologies for identification and serotyping, respectively (4).

Both isolates were cultured in Oxoid Nutrient agar overnight at 37°C and genomic DNA was extracted from a single colony using a QIAcube with QIAamp DNA Mini kit following the manufacturer’s instructions (Qiagen). The quality of genomic DNA was checked by QIAxpert (Qiagen), whereas DNA concentration was quantified using DeNovix (QFX Flurometer) for dsDNA high sensitivity kit. Genomic library was prepared using the Nextera XT kit (Illumina) according to the manufacturer’s protocol for multiplexed sequencing. Whole-genome short-reads sequencing was generated using MiSeq Illumina platform with the MiSeq V3 kit using 2 × 300 bp paired-end chemistry (600 cycles) (Illumina) according to manufacturer’s instructions. Raw data were demultiplexed using bcl2fastq tool version 2.20 (Illumina). The quality of the raw reads (FASTAQ files) was checked using FastQC tool version 0.11.9 (5). Genome sequences were assembled using combined reference-guided/*de novo* assembly with SeqMan NGen software (version 117.3.0.57; DNASTAR, Madison, WI). The genome sequences for both isolates were annotated by utilizing Rapid Annotation using Subsystem Technology (RAST version 2.0) (6–8). Multilocus sequence typing (MLST) was determined using MLST version 2.0.4 (9), where both isolates were assigned to ST548. In brief, the number of reads in total were 1939960 and 1533894 for Salmonella strains SA49317 and SA49319, respectively, presenting 101 and 89 total contigs (N50 value of 2720087 and 194754) distributed in genomes of 5,205,268 bp and 5,056,793 bp in size, respectively, with a G+C content of 52.1% (for both) with an average sequencing coverage depth of 80X. The antimicrobial Resistance genes were analyzed using ResFinder version 2.1 (10) and CARD version 4.0 tools (11) with a minimum percent identity at 90% and 60% for minimum query length. The results revealed that both strains carried *mcr-*1.1 gene. In addition, *sul2*, *tetA*, *bla_TEM-1B_*, *qnrB19*, *aac(6')-Iaa*, *aac(3)-IV*, and *floR* resistance genes were found in strain SA49317, whereas SA49319 strain had *sul2*, *tetA*, *bla_TEM-1B_*_,_ *qnrB19*, and *aac(6')-Iaa* resistance genes.

Plasmid replicons typing were identified using PlasmidFinder version 1.43 with a minimum percent identity at 95% and 60% for minimum query length (12). Default parameters were used for all software unless otherwise specified. Four plasmids were found in both isolates: Col(pHAD28), IncC, IncFII(S), and IncI1-I(Alpha).

In summary, we report draft genome sequences of 2 colistin-resistant Salmonella enterica serovar Minnesota strains belonging to ST548.

### Data availability.

This whole-genome project has been deposited in NCBI GenBank under BioProjects PRJNA844381 (for SA49317, accession number JANFCT000000000.1) and PRJNA844392 (for SA49319, accession number JANFCU000000000.1). The Illumina raw reads have been deposited at the Sequence Read Archive (SRA) under accession numbers SRR19759901 (for SA49317) and SRR19759701 (for SA49319).
